# Response of Pediatric Choroidal Neovascularization to Anti-Vascular Endothelial Growth Factor

**DOI:** 10.7759/cureus.20195

**Published:** 2021-12-06

**Authors:** Sunil Ruparelia, Aishwarya Sundaram, Mishari Dahrab, Chris Symonds, Alan Cruess

**Affiliations:** 1 Department of Ophthalmology, Faculty of Medicine, Dalhousie University, Halifax, CAN; 2 Department of Ophthalmology, Izaak Walton Killam Health Center, Halifax, CAN; 3 Department of Ophthalmology, Saint John Regional Hospital, Saint John, CAN

**Keywords:** ophthalmology, retina, pediatric ophthalmology, anti-vegf, choroidal neovascular membrane

## Abstract

Choroidal neovascularization (CNV) is a rare condition in children but poses a substantial threat to vision. Anti-vascular endothelial growth factor (anti-VEGF) therapy is commonly used in the pediatric population to treat retinopathy of prematurity. However, the use of anti-VEGF is less common for childhood CNV due to the rarity of CNV in the pediatric population. We report the case of a 10-year-old male presenting with an idiopathic choroidal neovascular membrane. Following a relapse of subretinal fluid after photodynamic therapy, anti-VEGF (bevacizumab) was injected and resulted in remission of the neovascular membrane and improved visual outcome. Further studies are required to elucidate the long-term outcomes associated with the use of anti-VEGF in pediatric patients.

## Introduction

Choroidal neovascularization (CNV) is a cause of substantial vision loss in both children and adolescents, particularly in cases presenting with subfoveal neovascularization. Although CNV most often affects adults, the impact on the pediatric population is devastating due to social and educational factors and the greater number of disability-adjusted life years [1]. Pediatric CNV is most often idiopathic, or occurs rarely secondary to inflammatory conditions, infections, trauma, or retinal dystrophy [2]. Clear guidelines for the management of pediatric CNV are somewhat limited due to a lack of randomized clinical trials [2]. However, an option becoming increasingly viable is the usage of a class of molecules known as vascular endothelial growth factor (VEGF) inhibitors, which act to inhibit neovascularization. Anti-VEGF therapy has been shown to improve vision in a number of pediatric ocular conditions involving pathogenic angiogenesis, including retinopathy of prematurity, diabetic retinopathy, and new blood vessel formation in response to retinal vascular occlusion [3]. However, the use of anti-VEGF therapy in pediatric choroidal neovascular membranes (CNVMs) is less commonly seen due to the rarity of CNV children. 

## Case presentation

A 10-year-old male presented to his local eye clinic with complaints of distortion in the right eye. Past ocular history was unremarkable. The patient was adopted, and therefore family ocular and early medical history were unknown. However, there was no known history of trauma. On examination, Snellen visual acuity was 20/32 in the right eye. The macula in the right eye appeared to have a centrally elevated foveal hyperpigmented area. The patient was referred to a tertiary referral center and was seen within the week.

**Figure 1:**
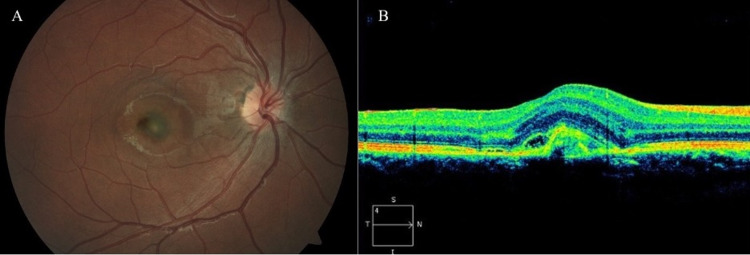
Fundus photo (A) showing subfoveal choroidal neovascular membrane and optical coherence tomography (B) demonstrating the presence of subretinal and intraretinal fluid.

When seen at the tertiary care center, retinal examination demonstrated edema with an underlying hyperpigmented membrane (Figure 1A) and optical coherence tomography (OCT) confirmed the presence of subretinal and intraretinal fluid with an apparent CNVM (Figure 1B).

**Figure 2:**
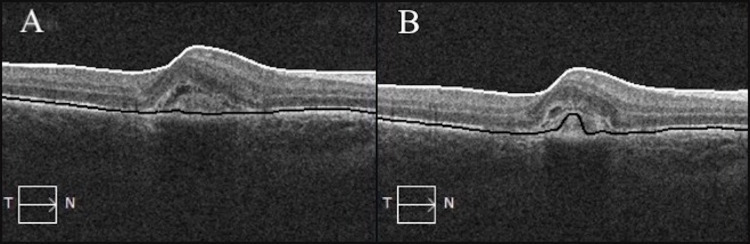
Pre-photodynamic therapy with CRT 350 µm (A) and post-photodynamic therapy with CRT 330 µm (B) of the right eye. CRT, central retinal thickness.

Initial bloodwork for histoplasmosis and toxoplasmosis titers were negative. Diode verteporfin photodynamic therapy (V-PDT) was applied as initial therapy. Three weeks post-PDT, visual acuity in the right eye was 20/40 and OCT showed a decrease in the overall thickness and a decrease in subretinal fluid in the area of the scar (Figure 2).

**Figure 3:**
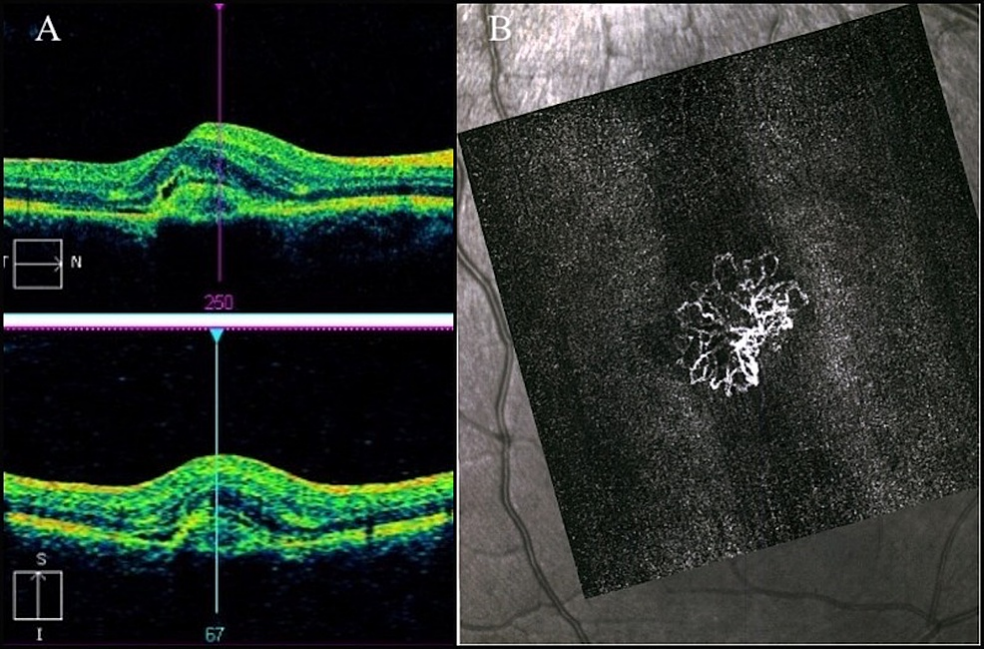
Pre-bevacizumab optical coherence tomography with CRT 409 µm (A) and optical coherence tomography angiography (B) of the right eye. CRT, central retinal thickness.

However, increased subretinal fluid and intraretinal fluid was noted three months later. The CNVM was deemed active, which was confirmed by OCT (Figure 3A) and OCT angiography (Figure 3B). At this time, the patient received 0.625 mg/0.025 mL intravitreous bevacizumab in the affected eye.

**Figure 4:**
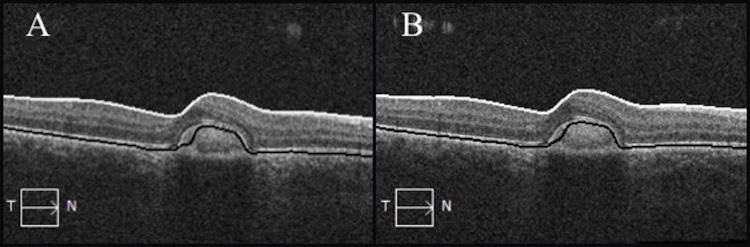
Optical coherence tomography 10 months post-bevacizumab with CRT 289 µm (A) and 16 months post-bevacizumab with CRT 287 µm (B) of the right eye. CRT, central retinal thickness.

Three weeks after injection, the patient reported improved vision in the right eye, with 20/30 Snellen visual acuity. A macular scar was noted, but there were no signs of retinal hemorrhage or edema. OCT of the right eye 10 months and 16 months after injection demonstrated persistent decrease in the overall retinal thickness and absence of subretinal fluid (Figure 4).

## Discussion

There are several notable differences that exist in the management of CNVMs when treating pediatric patients as compared to adults. Neovascular age-related macular degeneration and progressive myopia (myopic choroidal neovascularization) are among the most common etiologies in adult populations. Conversely, the differential diagnosis in pediatric CNV includes idiopathic CNV (most common), or, more rarely, CNV secondary to inflammatory conditions, infections, trauma, or retinal dystrophy [2]. Despite different etiologies, treatment options available to manage pediatric CNV are similar to those used in adults.

As illustrated in this case, V-PDT or intraocular anti-VEGF injection may be used as monotherapy or in combination to manage pediatric CNVM. Fewer anti-VEGF injections are typically required to manage pediatric neovascularization as compared to adults, with improvements seen after as few as two injections [4]. Further, a randomized clinical trial investigating pediatric dosing requirements of anti-VEGF for treatment of a variety of exudative conditions showed that 63% of cases resolved after a single injection, with the remaining cases resolving after two injections. With the exception of neonates, the dose of VEGF inhibitors used in the pediatric population is typically 0.625 mg/0.025 mL [5]. However, it is very possible that small children may achieve favorable outcomes with reduced dosing. This is suggested as a topic for further study, as reduced dosing would lower the risk of possible side effects. 

There are several benefits to anti-VEGF injections that may make them the standard first-line treatment option in future practice. Despite the proven efficacy of PDT for the treatment of neovascularization, compliance during treatment can be an issue, particularly with pediatric patients [6]. Furthermore, anti-VEGF therapy may provide more favorable visual outcomes than other treatment options, particularly with subfoveal CNV, due to reduced damage to the retina and choroid [7].

There is ongoing debate on the long-term side-effects of anti-VEGF therapy. In adults, some studies have suggested that repeated injections may lead to a sustained increase in intraocular pressure, which could necessitate further intervention. In a recent study, it was demonstrated that an increased risk of requiring glaucoma surgery was seen in patients who received seven or more injections of bevacizumab [8]. However, these side-effects may pose less of a threat in the pediatric population, as fewer injections are generally required to resolve neovascularization and edema. It is postulated that this is likely due to the healthier retinal pigmented epithelium pumps seen in younger patients [4]. Some studies suggest that there are side-effects of anti-VEGF injections that are unique to the pediatric patient population. However, most of these studies refer specifically to neonatal injections, and involve uptake of the inhibitors of angiogenesis into other tissues. One such study suggests that vascular leakage of injected bevacizumab in neonates could lead to hypoxia of the surrounding brain tissues and result in neurological disorders [9]. Using similar reasoning, another case report suggests that systemic uptake of VEGF inhibitors can lead to necrotizing enterocolitis in neonates after intravitreal bevacizumab injections [10]. It may be that the older pediatric population benefit from requiring fewer injections than their adult counterparts, while also being less susceptible to systemic effects than are neonates. Despite anti-VEGF therapy being well tolerated in this case, further research should seek to better understand the possible long-term effects on pediatric patients.

## Conclusions

CNV is a rare condition in the pediatric population. As a result, there is limited literature describing the use of anti-VEGF injections in the therapeutic management of pediatric CNV. This case describes a successful management approach to pediatric CNV, demonstrating lasting remission of the neovascular membrane and improved visual outcome with anti-VEGF therapy.
